# Ambient Air Pollution Associated with Suppressed Serologic Responses to *Pneumocystis jirovecii* in a Prospective Cohort of HIV-Infected Patients with *Pneumocystis* Pneumonia

**DOI:** 10.1371/journal.pone.0080795

**Published:** 2013-11-13

**Authors:** Robert J. Blount, Kpandja Djawe, Kieran R. Daly, Leah G. Jarlsberg, Serena Fong, John Balmes, Robert F. Miller, Peter D. Walzer, Laurence Huang, on behalf of the International HIV-associated Opportunistic Pneumonias (IHOP) Study.

**Affiliations:** 1 Division of Pulmonary and Critical Care Medicine, San Francisco General Hospital, University of California San Francisco, San Francisco, California, United States of America; 2 Department of Internal Medicine, Division of Infectious Diseases, University of Cincinnati, Cincinnati, Ohio, United States of America; 3 Department of Environmental Health, Division of Epidemiology and Biostatistics, University of Cincinnati, Cincinnati, Ohio, United States of America; 4 Veterans Administration Medical Center, Cincinnati, Ohio, United States of America; 5 School of Public Health, University of California Berkeley, Berkeley, California, United States of America; 6 Research Department of Infection and Population Health, Institute of Epidemiology and Healthcare, University College London, London, United Kingdom; 7 Department of Infectious and Tropical Diseases, London School of Hygiene and Tropical Medicine, London, United Kingdom; 8 HIV/AIDS Division, San Francisco General Hospital, University of California San Francisco, San Francisco, California, United States of America; Vanderbilt University, United States of America

## Abstract

**Background:**

Ambient air pollution (AAP) may be associated with increased risk for *Pneumocystis pneumonia* (PCP). The mechanisms underlying this association remain uncertain.

**Objectives:**

To determine if real-life exposures to AAP are associated with suppressed IgM antibody responses to *P. jirovecii* in HIV-infected (HIV+) patients with active PCP, and to determine if AAP, mediated by suppressed serologic responses to *Pneumocystis*, is associated with adverse clinical outcomes.

**Methods:**

We conducted a prospective cohort study in HIV+ patients residing in San Francisco and admitted to San Francisco General Hospital with microscopically confirmed PCP. Our AAP predictors were ambient air concentrations of particulate matter of < 10 µm in diameter (PM_10_) and < 2.5 µm in diameter (PM_2.5_), nitrogen dioxide (NO_2_), ozone (O_3_), and sulfur dioxide (SO_2_) measured immediately prior to hospital admission and 2 weeks prior to admission. Our primary outcomes were the IgM serologic responses to four recombinant *P. jirovecii major* surface glycoprotein (Msg) constructs: MsgC1, MsgC3, MsgC8, and MsgC9.

**Results:**

Elevated PM_10_ and NO_2_ exposures immediately prior to and two weeks prior to hospital admission were associated with decreased IgM antibody responses to *P. jirovecii* Msg. For exposures immediately prior to admission, every 10 µg/m^3^ increase in PM_10_ was associated with a 25 to 35% decrease in IgM responses to Msg (statistically significant for all the Msg constructs), and every 10 ppb increase in NO_2_ was associated with a 19-45% decrease in IgM responses to Msg (statistically significant for MsgC8 and MsgC9). Similar findings were seen with exposures two weeks prior to admission, but for fewer of the Msg constructs.

**Conclusions:**

Real life exposures to PM_10_ and NO_2_ were associated with suppressed IgM responses to *P. jirovecii* Msg in HIV+ patients admitted with PCP, suggesting a mechanism of immunotoxicity by which AAP increases host susceptibility to pulmonary infection.

## Introduction

Greater than three million deaths per year are attributable to ambient air pollution (AAP), and AAP is the 9^th^ leading factor contributing to worldwide burden of disease according to the 2010 Global Burden of Disease Study [1]. AAP is well established as a risk factor for a wide array of cardiopulmonary diseases, and there is emerging data that it is associated with pulmonary infections. For instance, a number of observational studies have found AAP to be associated with increased emergency department visits and hospital admissions for community acquired pneumonia [2-12]. AAP may also contribute to the morbidity of other pulmonary infections, although data in this area is limited. It is also unclear if the effects of AAP are present and perhaps magnified in immunosuppressed populations, such as those with HIV, who are already at increased risk for pulmonary infections [13,14].

*Pneumocystis* pneumonia (PCP) continues to contribute to substantial morbidity and mortality in immunosuppressed children and adults worldwide [15], and a better understanding of environmental risk factors contributing to PCP incidence and disease severity is needed. In one recent case-crossover study Djawe et al. observed an association between high AAP levels and increased hospital admissions for PCP among HIV-infected (HIV+) patients [16]. The underlying mechanisms for this increased susceptibility for PCP hospitalizations with AAP exposure remain unknown, but mechanisms of AAP induced immunotoxicity seem most plausible. As *Pneumocystis* is extremely difficult to grow in culture, we have developed an enzyme-linked immunosorbent assay (ELISA) using recombinant *Pneumocystis jirovecii major* surface glycoprotein (Msg) to assess serologic responses to *P. jirovecii* [17-21]. We selected this assay for the current study because it has been validated in diverse cohorts worldwide [22,23] and has been informative in assessing immunologic responses to *Pneumocystis* in those with HIV infection [24].

The mechanisms by which ambient air pollution increases susceptibility to pulmonary infection are not well defined. Single pollutant controlled exposure studies using cells, animals and human subjects have found that ambient air pollutants alter innate lung immunity at multiple levels [25] including altered mucociliary function [26], respiratory epithelial cell dysfunction [27,28], impaired alveolar macrophage phagocytosis [29-32], and surfactant protein A and D dysfunction [33]. However, the effects of AAP on humoral immunity and serologic responses to pulmonary infection, and the immunotoxic effects of real-life exposures to AAP remain unclear.

To address these areas of uncertainty we proposed two hypotheses for this study. First, real-life exposures to AAP suppress IgM antibody responses to *P. jirovecii* Msg, and second, AAP exposures, mediated by suppressed serologic responses to *P. jirovecii* Msg, are associated with adverse clinical outcomes. To test these hypotheses we conducted a prospective cohort study of HIV+ patients residing in San Francisco and hospitalized at San Francisco General Hospital with confirmed PCP.

## Methods

### Study population

From June 2000 to September 2007 we enrolled consecutive HIV+ adults (age > 18 years) hospitalized at San Francisco General Hospital with suspected PCP into a longitudinal cohort [34]. From this cohort, those with a definitive microscopic diagnosis of PCP and residence within the San Francisco city limits were eligible for inclusion in our prospective study. We excluded those with prior enrollment into the cohort and those without an available serologic specimen within 2 days of hospital admission. Hospital admission criteria for PCP included a supplemental oxygen requirement and/or poor oral intake, while discharge criteria included oxygen saturations > 90% while breathing room air and adequate oral intake.

### Clinical and demographic data collection

After obtaining written, informed consent, we administered a standardized questionnaire to all participants, followed them throughout their hospitalization, and performed chart abstraction upon discharge from the hospital to obtain the following clinical data: age, sex, race/ethnicity, residential address, cigarette smoking status, prior history of PCP, antiretroviral medication and PCP prophylaxis, CD4 cell count, HIV viral load, length of hospital stay, admission to intensive care unit, intubation, and in-hospital mortality. 

### Ambient air pollutant measurements

We evaluated criteria pollutants most implicated in cardiopulmonary disease: particulate matter of < 10μm (PM_10_) and < 2.5μm in diameter (PM_2.5_), nitrogen dioxide (NO_2_), ozone (O_3_), and sulfur dioxide (SO_2_). The Bay Area Air Quality Management District monitors air pollutant concentrations in San Francisco at a centrally located monitoring station on 10 Arkansas Street for U.S. Environmental Protection Agency (EPA) regulatory compliance purposes. We used these publically available data to assign AAP exposures for each participant immediately prior to hospital admission and 2 weeks prior to admission. We chose these two exposure periods a priori based on biological plausibility of the timing of these exposures being associated with serologic responses to *P. jirovecii* at admission. For each period, we calculated the average exposure over 3 days. For the period immediately prior to admission, we averaged exposures from the day of admission, 1 day prior to admission, and 2 days prior to admission (days 0, -1, and -2). For the period 2 weeks prior to admission, we averaged exposures from days 14, 15, and 16 prior to admission (days -14, -15, and -16). The air pollutant metrics used in the analysis were as follows: daily mean PM_10_ and PM_2.5_ concentrations (μg/m^3^), daily maximum 1-hour NO_2_ concentrations in parts per billion (ppb), daily maximum 8-hour O_3_ concentrations (ppb), and daily maximum 1-hour SO_2_ concentrations (ppb). 

### Recombinant antigens

Using a λgt11 clone of Msg gene obtained from human infected lung as our template, we amplified the gene segment Msg_2015-3332_ (subscript denotes number of nucleotides) using PCR with AmpliTaq enzyme (Applied Biosystems, Carlsbad, CA, USA) [18]. We then inserted the amplified gene segment into the pET30 *E. coli* expression system (Novagen, Madison, WI, USA) to generate MsgC1 recombinant protein. We purified this protein by affinity chromatography. Using a similar process and *P. jirovecii*-infected human lung as our template, we generated MsgC3, MsgC8, and MsgC9 recombinant proteins from the same gene region as for MsgC1 [20]. These MsgC constructs are representative of the most antigenically conserved carboxyl-terminus region of the major surface glycoprotein [17]. 

### IgM ELISA

We collected serum from each subject within two days of hospital admission and stored the sera at -80°C until shipment to University of Cincinnati for IgM ELISA analysis. Using a previously described protocol developed in our lab [21], we measured serum IgM responses to the four recombinant MsgC constructs; MsgC1, MsgC3, MsgC8, and MsgC9. We tested subject serum specimens and standard reference sera against the recombinant Msg constructs, using phosphate-buffered saline (PBS) without Msg as the negative control. We corrected the reactivity of each serum specimen to Msg by subtracting the reactivity of each serum specimen to PBS (mean optical density (OD) with Msg – mean OD without Msg) and quantified the results using methods described by Bishop and Kovacs [35]. We prepared a standard serum with specificity for each Msg construct by mixing the sera from 4 to 6 specimens with high reactivity for the specific construct. We selected these specimens from banks of sera from blood donors and HIV+ patients. The standard pool for each Msg construct was defined as having a value of 100 U in 100 μl of a 1:100 dilution. We used the same standard pools throughout the study. From the standard pool, we generated a standard curve for each Msg construct on each day the assay was used. We used this curve to calculate the units of reactivity to the Msg construct. We diluted test serum samples at 1:100 to fit the linear portion of the standard curves. Taking into account the dilution, we then calculated units of reactivity.

### Ethics approval

The study was approved by the institutional review boards at the University of California San Francisco and the University of Cincinnati.

### Statistical analysis

We evaluated associations between ambient air pollutant concentrations and IgM antibody responses to Msg constructs using multivariable tobit regression analysis for left censored data. Our predictors were exposures to PM_10_, PM_2.5_, NO_2_, O_3_, and SO_2_. Our primary outcome variables were log-transformed serum IgM antibody levels (in units of reactivity) to each Msg construct (MsgC1, MsgC3, MsgC8, and MsgC9). We log-transformed outcome variables to best conform to regression model assumptions. We tested for the effect modification of CD4 cell count and active cigarette smoking using interaction terms in the tobit regression analysis. We selected these variables as potential effect modifiers based on prior literature [24,36] and the biologic plausibility of their interactions with air pollutants in their associations with IgM responses to Msg constructs. Our pre-specified criteria for reporting effect modification were a p < 0.20 and a difference between stratum specific regression coefficients of at least 20%. We scaled and exponentiated regression coefficients to express logged outcomes as percent change in IgM response to each Msg construct per 10 unit increase in air pollutant concentration. We also used multivariable tobit regression analysis to evaluate the associations between clinical factors (CD4 cell count, viral load, and active cigarette smoking) and IgM responses, adjusting for a priori selected potential confounders (PM_10_, NO_2_, smoking, and CD4 cell count). 

 We evaluated associations between air pollutant (PM_10_, PM_2.5_, NO_2_, O_3_, and SO_2_) concentrations and clinical outcomes using multivariable linear regression for the continuous outcome (length of hospital stay) and logistic regression for the binary outcomes (ICU admission, intubation, and in-hospital mortality), evaluating for effect modification of log-transformed CD4 cell count and active cigarette smoking as detailed above.

To evaluate the association of IgM responses with clinical outcomes, we used linear regression for the continuous outcome (length of hospital stay) and logistic regression for the binary outcomes (ICU admission, intubation, and in-hospital mortality), adjusting for log-transformed CD4 cell count, active cigarette smoking, and PCP prophylaxis as a priori selected potential confounders. Finally, we performed a mediation analysis to determine the indirect effects of IgM responses on clinical outcomes compared with the direct effects of air pollutants on clinical outcomes.

## Results

### Subjects and ambient air pollution

**Figure 1:**
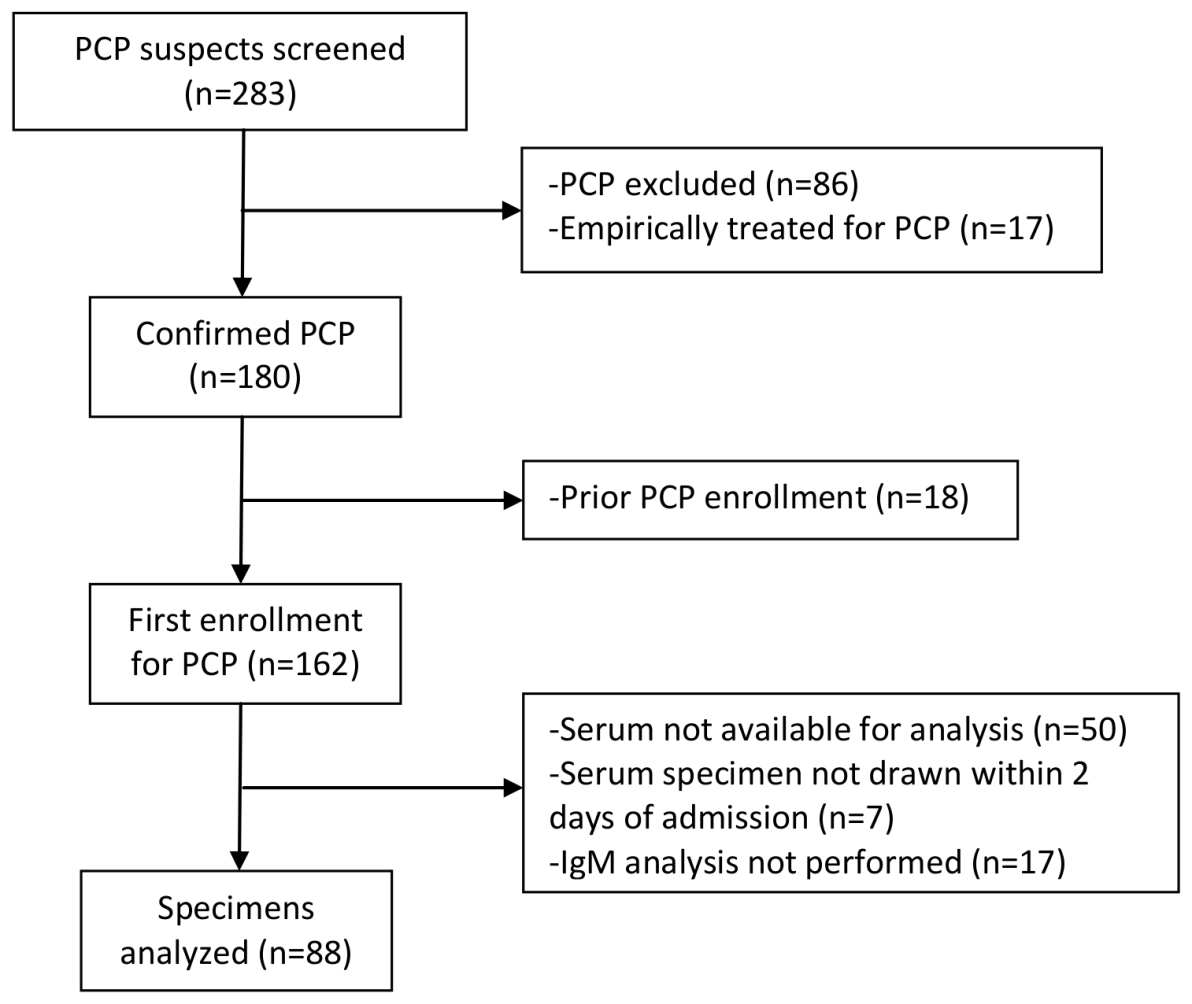
Participant enrollment.

We enrolled 283 consecutive HIV+ patients with suspected PCP into the larger cohort study. A definitive microscopic diagnosis of PCP was made in 180 of these patients, 162 of whom were being enrolled into the cohort with PCP for the first time (Figure 1). Of these, 88 subjects with PCP had serum available within 2 days of admission on which IgM serologic analysis was performed. The majority were male (n=77, 88%), white (n=48, 55%), and active cigarette smokers (n=59, 67%) (Table 1). Only 10 (11%) were taking antiretroviral therapy (ARV) and eight (9%) taking PCP prophylaxis at the time of admission. The median CD4 cell count was 33 cells/μl (interquartile range 10-77 cells/μl) and the mean log HIV RNA level was 11.3 copies/ml (standard deviation ± 2.0 copies/ml), reflecting a severely immunosuppressed cohort with uncontrolled viremia. Overall, nine patients required ICU admission (10%) and four (5%) died during the hospitalization. Among first-time enrollees with confirmed PCP (n=162), we compared those patients excluded (n=74) to those included in the study (n=88) and found no statistically significant differences in baseline clinical characteristics or clinical outcomes (data not shown).

**Table 1:** Cohort demographics and clinical characteristics (n=88).

**Characteristic**		**n** (%)*
Median age (IQR)		40 (36–46)
Male sex		77 (88)
Race/ethnicity:	White	48 (55)
	Black	29 (33)
	Other	11 (13)
Active tobacco smoker		59 (67)
Homeless		16 (18)
ARV use		10 (11)
PCP prophylaxis use		8 (9)
Median CD4 cell count, cells/μl (IQR)		33 (10–77)
Log viral load ± SD		11.3 ± 2.0
Median length of stay, days (IQR)^†^		7 (5-10)
Admission to ICU		9 (10)
Intubation		7 (8)
In-hospital mortality		4 (5)

SD indicates one standard deviation; IQR, interquartile range; ICU, intensive care unit; ARV, antiretroviral medication.

* Results in parentheses represent percentages unless otherwise indicated.

^†^ Among those who survived hospitalization (n=84)

**Figure 2:**
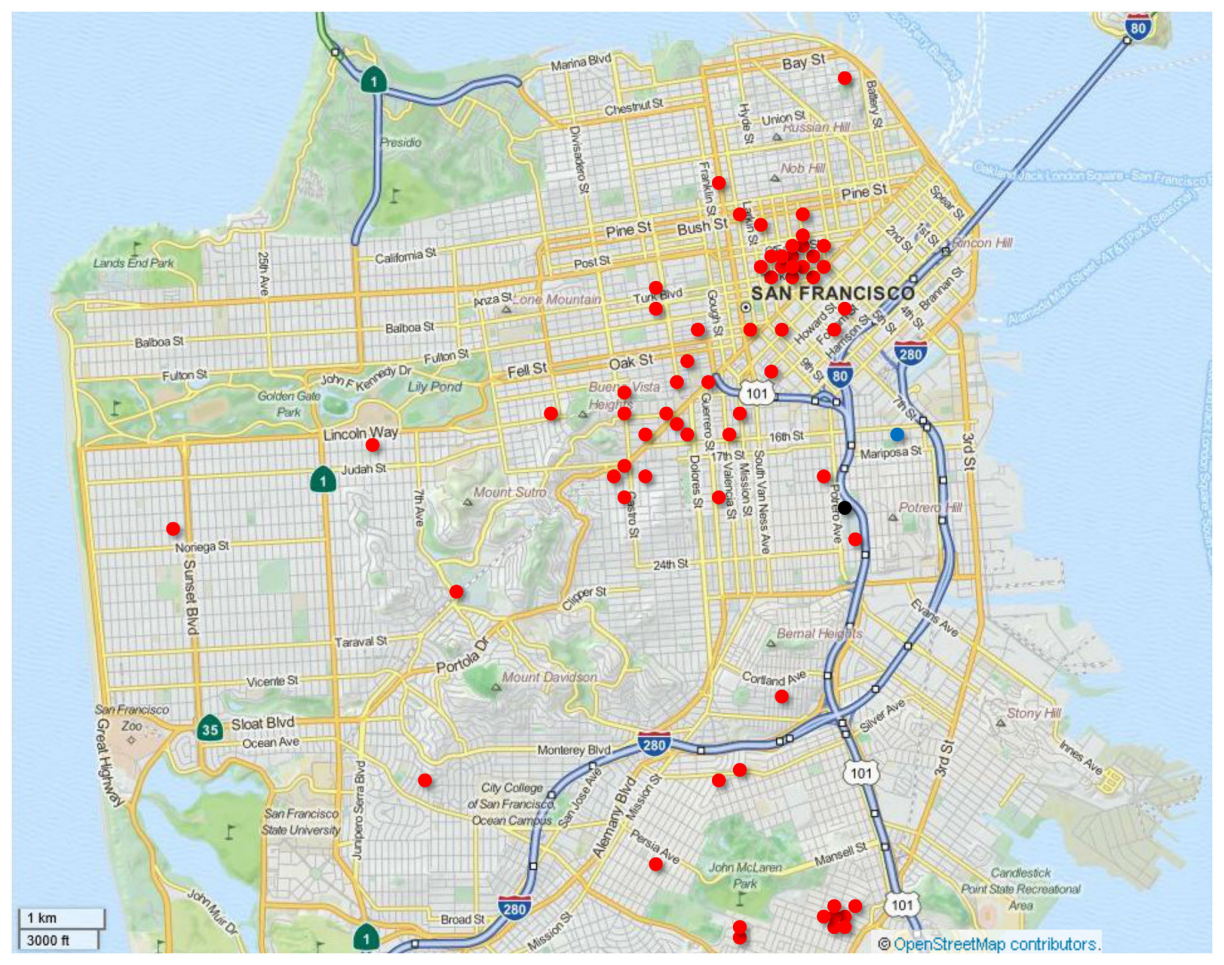
Map of San Francisco showing geographic distribution of participants (n=65).*. Legend: Blue circle— EPA compliance air quality monitoring station; Black circle—study site (San Francisco General Hospital); Red circles—residential area for each participant. *Residential addresses not available for 23 subjects. Map created at www.openstreetmap.org. Cartography licensed as CC-BY-SA.

**Figure 3:**
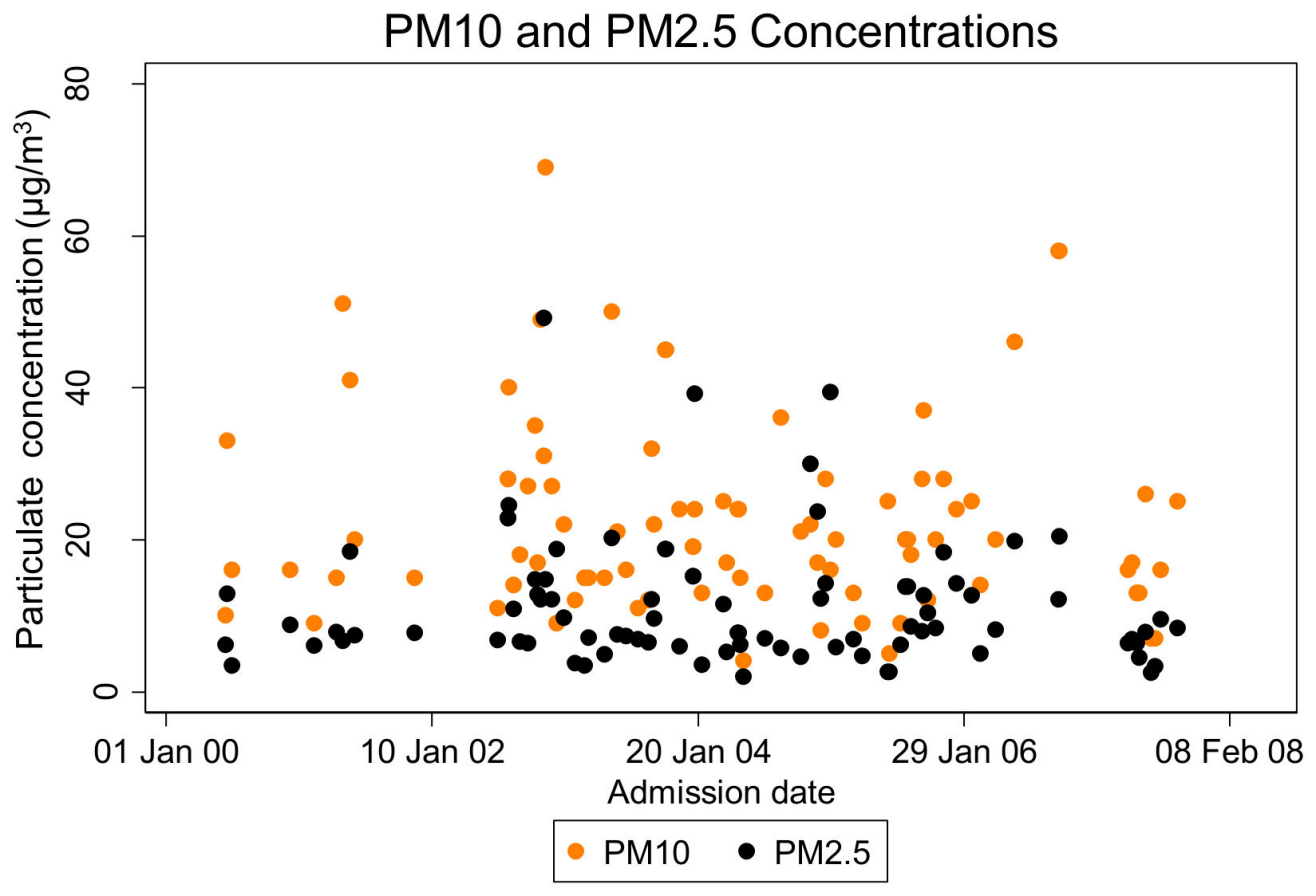
Three-day average particulate concentrations immediately prior to each participant’s hospital admission.

**Figure 4:**
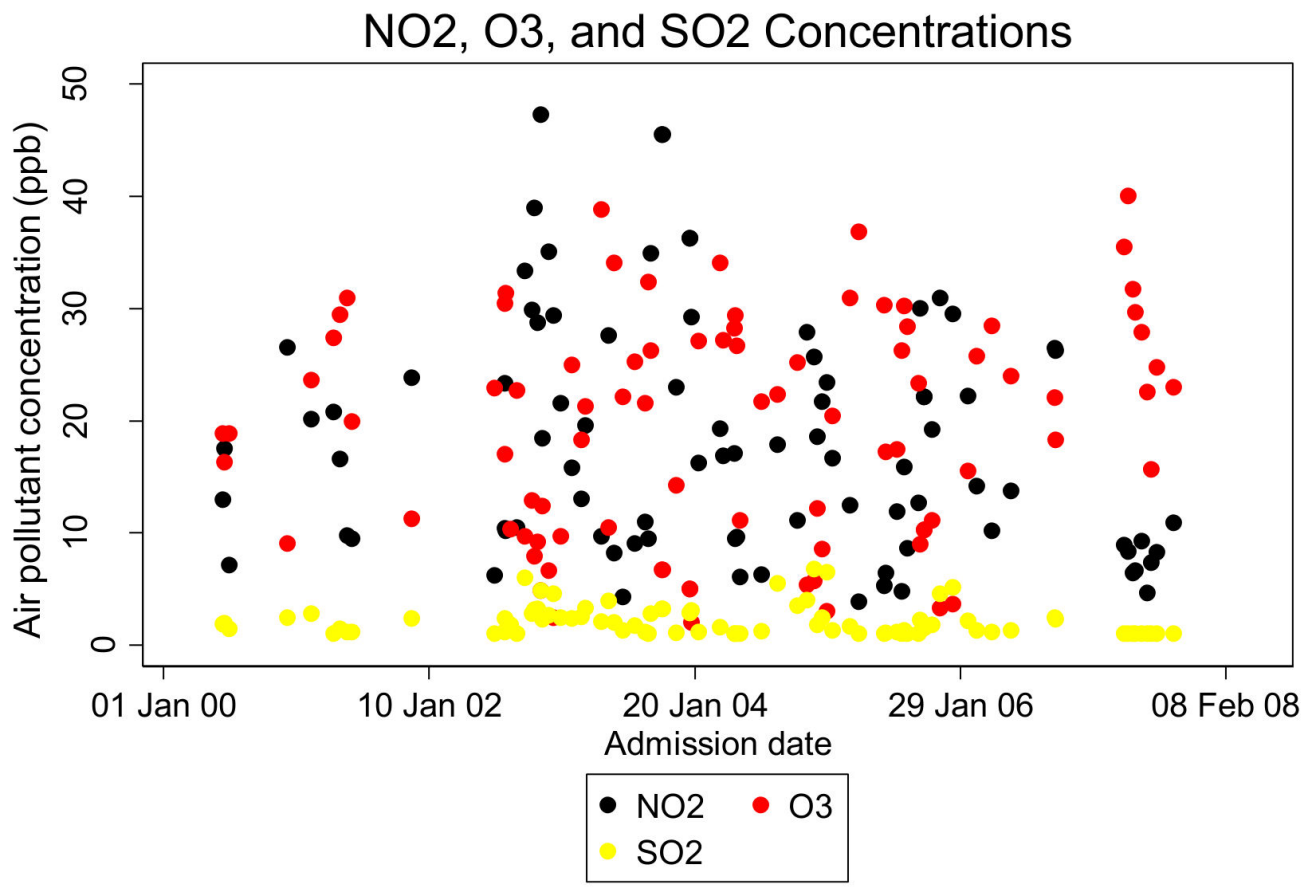
Three-day average NO_2_, O_3_, and SO_2_ concentrations immediately prior to each participant’s hospital admission.

All study participants resided within the San Francisco city limits. Specific addresses were available for 65 subjects (74%) (Figure 2). All of these resided within 9 km of the monitoring station, with the majority (n=36, 55%) residing within a 3km radius of the station. Three-day average exposures to ambient air pollutants immediately prior to and 2 weeks prior to hospital admission varied considerably over the study period (Figures 3, 4), and were much less than the maximum allowable concentrations as defined by National Ambient Air Quality Standards (NAAQS) (Table 2). SO_2_ concentrations were particularly low throughout the study period. 

**Table 2:** Mean air pollution exposure concentrations for study participants compared with NAAQS (n=88).

		**Pollutant concentration ± SD**		
**Pollutant**	**Averaging time**	Immediately prior to admission*	2 weeks prior to admission^†^	NAAQS
PM_10_ (μg/m^3^)	24-hour	22.6 ± 12.9	21.6 ± 11.1	150
PM_2.5_ (μg/m^3^)	24-hour	11.3 ± 8.3	9.7 ± 7.5	35
NO_2_ (ppb)	1-hour	18.0 ± 10.5	15.9 ± 8.3	100
O_3_ (ppb)	8-hour	19.4 ± 9.9	21.0 ± 7.9	75
SO_2_ (ppb)	1-hour	2.1 ± 1.4	1.9 ± 1.2	75

SD indicates one standard deviation; NAAQS, National Ambient Air Quality Standards; PM_10_, particulate matter of < 10 µm in diameter; PM_2.5_, particulate matter of < 2.5 µm in diameter; NO_2_, nitrogen dioxide; O_3_, ozone; SO_2_, sulfur dioxide.

* 3-day average: the day of admission, 1 day prior to admission, and 2 days prior to admission

^†^ 3-day average: days 14, 15, and 16 prior to admission

## Association between AAP and IgM

Elevated 3-day average ambient air concentrations of PM_10_ and NO_2_ immediately prior to hospital admission were associated with decreased IgM responses to all of the analyzed Msg constructs (Table 3). For every 10 μg/m^3^ increase in PM_10_ there was a 25% decrease in IgM response to MsgC1 (p=0.002), a 35% decrease in IgM to MsgC3 (p=0.009), a 33% decrease in IgM to MsgC8 (p=0.009), and a 25% decrease in IgM response to MsgC9 (p=0.02). We found a similar trend for NO_2_: elevated ambient air concentrations of NO_2_ were associated with decreased IgM responses to all the Msg constructs. These results reached statistical significance for MsgC8 and MsgC9. For every 10 ppb increase in NO_2_ there was a 45% decrease in IgM response to MsgC8 (p<0.001) and a 44% decrease IgM response to MsgC9 (p<0.001). On the other hand, we found no statistically significant associations or consistent trends between PM_2.5_, O_3_, or SO_2_ immediately prior to hospital admission and IgM responses to Msg (data not shown).

**Table 3:** Percent change in IgM responses to Msg constructs per 10 unit increase in pollutant exposure immediately prior to hospital admission and 2 weeks prior to admission (n=88).

		**MsgC1**			**MsgC3**			**MsgC8**			**MsgC9**		
**Pollutant** (units)	**Exposure period** (days prior to admission)*	%	(95%CI)	p value	%	(95%CI)	p value	%	(95%CI)	p value	%	(95%CI)	p value
**PM_10_** (μg/m^3^)	0 to -2	-25	(-41 to -9.4)	0.002	-35	(-61 to -9.1)	0.009	-33	(-57 to -8.5)	0.009	-25	(-46 to -4.1)	0.02
	-14 to -16	0.64	(-25 to 26)	0.96	-45	(-73 to -18)	0.002	-34	(-62 to -6.9)	0.02	-22	(-49 to 4.0)	0.10
**NO_2_** (ppb)	0 to -2	-19	(-41 to 3.2)	0.09	-30	(-62 to 3.4)	0.08	-45	(-68 to -23)	<0.001	-44	(-63 to -24)	<0.001
	-14 to -16	-15	(-44 to 14)	0.30	-41	(-77 to -5.3)	0.03	-63	(-84 to -42)	<0.001	-37	(-65 to -10)	0.008

Msg indicates major surface glycoprotein; CI, confidence interval; PM_10_, particulate matter < 10 μm in diameter; NO_2_, nitrogen dioxide.

* Where the day of hospital admission is indicated by “day 0”

Similarly, we found that elevated 3-day average concentrations of PM_10_ and NO_2_ in San Francisco 2 weeks prior to admission were associated with decreased IgM responses to MsgC, but were statistically significant for fewer MsgC constructs (Table 3). Every 10 μg/m^3^ increase in PM_10_ predicted a 45% decrease in IgM response to MsgC3 (p=0.002) and a 34% decrease in IgM to MsgC8 (p=0.02). Likewise, every 10 ppb increase in NO_2_ predicted a 41% decrease in IgM response to MsgC3 (p=0.03), a 63% decrease in IgM response to MsgC8 (p<0.001) and a 37% decrease in IgM response to MsgC9 (p=0.008). As with exposures immediately prior to admission, we did not find statistically significant associations between PM_2.5_, O_3_, or SO_2_ two weeks prior to admission and IgM responses to Msg.

### Effect of cigarette smoking on the association between AAP and IgM

Active cigarette smoking appeared to augment the immunosuppressive effects of PM_10_ and NO_2_ exposure 2 weeks prior to admission on IgM responses to Msg (data stratified by smoking status not shown). For every 10 μg/m^3^ increase in PM_10_ smokers experienced a larger decline in IgM responses to all the constructs compared with nonsmokers. This interaction was statistically significant for MsgC1 (p=0.04), MsgC3 (p=0.03), MsgC8 (p=0.01), and of borderline statistical significance for MsgC9 (p=0.07). A similar interaction was seen between cigarette smoking and NO_2_ exposure 2 weeks prior to admission. For every 10 ppb increase in NO_2_, smokers experienced a larger decline in IgM responses to all of the Msg constructs compared with nonsmokers. This interaction was statistically significant for MsgC8 (p=0.03) and MsgC9 (p=0.01). Smoking, on the other hand, did not appear to be directly associated with IgM responses in bivariate analyses (data not shown) or after adjusting for CD4 cell count, PM_10_, and NO_2_ (see Table 4). 

**Table 4:** Percent change in IgM responses to Msg constructs by clinical factors (n=88).*

	**MsgC1**			**MsgC3**			**MsgC8**			**MsgC9**		
**Clinical factors**	%	(95%CI)	p value	%	(95%CI)	p value	%	(95%CI)	p value	%	(95%CI)	p value
CD4 cell count^†^	12	(2.5 to 22)	0.02	9.4	(-7.3 to 26)	0.27	16	(1.5 to 31)	0.03	9.3	(-3.1 to 22)	0.14
Viral load^†^	-2.3	(-8.0 to 3.4)	0.42	-5.9	(-15 to 3.6)	0.22	-6.8	(-15 to 1.1)	0.09	-0.5	(-8.0 to 7.0)	0.90
Active smoking^#^	2.3	(-58 to 62)	0.94	34	(-105 to 172)	0.63	-18	(-89 to 52)	0.61	-9.8	(-79 to 59)	0.78

* Percent change in IgM responses to Msg constructs per 50% increase in CD4, 50% increase in viral load, or for active cigarette smoking status

^†^ Adjusted for active smoking, and PM_10_ and NO_2_ immediately and 2 weeks prior to admission

^#^ Adjusted for CD4 cell count, and PM_10_ and NO_2_ immediately and 2 weeks prior to admission

Indicators of more intact immunity were associated with increased IgM responses to Msg (Table 4). For every 50% increase in CD4 cell count there was a 9.3% to 16% increase in IgM responses to Msg, statistically significant for MsgC1 (p=0.02) and MsgC8 (p=0.03). On the other hand, for every 50% increase in viral load there was a non-statistically significant decrease in IgM responses to Msg of 0.5% to 6.8%.

### Association between AAP and clinical outcomes

Higher IgM responses to all the Msg constructs were associated with shorter length of hospital stay, with each 50% increase in IgM associated with a 0.36 to 0.90 day decrease in length of stay (Table 5). However, this was statistically significant only for MsgC8 (p=0.03). IgM responses to Msg were not associated with other clinical outcomes (ICU admission, intubation, or in-hospital mortality), nor were 3 day average air pollution exposures immediately and 2 weeks prior to hospital admission associated with length of stay, ICU admission, intubation, or in-hospital mortality. Similarly, the ambient air pollutants were not associated with other surrogates for PCP severity, such as serum lactate dehydrogenase (LDH). We also evaluated the association of each ambient air pollutant with CD4 cell count as the clinical outcome, and found no statistically significant associations between AAP and CD4 cell count (data not shown). 

**Table 5:** Change in length of hospital stay (days) per 50% increase in IgM response to Msg among those who survived to hospital discharge (n=84).*

**IgM response to Msg**		**Length of stay**		
Construct	Geometric mean, units (95% CI)	# days	(95%CI)	p value
MsgC1	14.2 (11.0–18.4)	-0.36	(-1.2 to 0.53)	0.42
MsgC3	3.8 (2.8–5.1)	-0.53	(-1.3 to 0.21)	0.16
MsgC8	3.5 (2.6–4.6)	-0.90	(-1.7 to -0.10)	0.03
MsgC9	5.7 (4.3–7.6)	-0.57	(-1.3 to 0.19)	0.14

* Adjusted for log CD4 cell count, active cigarette smoking, and PCP prophylaxis.

## Discussion

To our knowledge, this is the first prospective cohort study to evaluate the association of ambient air pollution exposures on serologic responses to *Pneumocystis jirovecii* and clinical outcomes in HIV+ patients hospitalized with confirmed PCP. We found that elevated ambient concentrations of PM_10_ and NO_2_ were associated with suppressed IgM responses to recombinant *Pneumocystis* major surface glycoprotein, and that this suppression of responses may be associated with increased length of hospital stay. Smoking appeared to augment the immunosuppressive effects of PM_10_ and NO_2_.

PM_10_ and NO_2_ were specifically associated with suppressed IgM responses, while PM_2.5_, O_3_, and SO_2_ were not. When designing this study, we hypothesized that particulate matter would play a significant role in altering serologic responses. Particle deposition in the lungs is size-dependent; so-called “fine” particles < 2.5 μm in aerodynamic diameter are inhaled to the alveolar level, whereas somewhat larger particles from 2.5 to 10 μm in diameter (the so-called “coarse” fraction) tend to deposit on lower airway mucociliary surfaces. As PM_10_ includes both fine and coarse particles it is possible that PM_10_ exerts its immunotoxicity in both airway and alveolar compartments. Coarse particles encountering bronchus associated lymphoid tissue, for instance, could impair antigen presenting cells, resulting in decreased activation of the humoral immune system and suppressed serologic responses, while fine particles could exert immunotoxic effects on alveolar macrophages, also resulting in impaired humoral activation. Periodic northeasterly (“Diablo”) winds bring coarse particulate matter of crustal origin into San Francisco. Such particulate matter can be highly bioactive. It is possible that the greater magnitude and variability in PM_10_ exposure afforded by these local weather patterns contributed to more significant findings for PM_10_ than PM_2.5_. Similarly, NO_2_ could be immunotoxic at the alveolar level or, freely diffusible, directly toxic to B lymphocytes. SO_2_ levels were clearly too low in this study to be implicated in immunologic derangement or clinical outcomes. San Francisco has exceedingly low SO_2_ pollution due to the absence of coal-powered power plants and industry in the city as well as California regulations requiring the use of low sulfur fuel. Ozone concentrations are also very low in San Francisco due to prevailing westerly winds that carry ozone precursors from motor vehicle emissions eastward across the Bay. PM_10_ and NO_2_ levels were statistically significantly associated with most, but not all, Msg constructs. It is unclear, for instance, why associations between NO_2_ and the Msg constructs MsgC8 and MsgC9 were more statistically significant than for MsgC1 and MsgC3.

We selected the timing of the periods during which air pollutant exposures were assigned to subjects based on a priori hypothesized mechanisms of immunotoxicity. We posited that pollutants with capacity to cross the alveolar capillary interface into blood circulation (e.g., NO_2_) could have early impact on B cell production of *Pneumocystis jirovecii* specific IgM. On the other hand, we hypothesized that coarse particulate matter, as mentioned above, would exert its effects predominantly via impaired antigen presentation and delayed humoral activation, an effect that might take 1 to 2 weeks to fully realize. Supportive of these mechanistic hypotheses, we found PM_10_ and NO_2_ exposures both immediately prior to hospital admission and 2 weeks prior to admission to be associated with suppressed IgM responses. These were 3-day averaged exposures during the two periods, and it remains unclear whether peak exposures might have independent associations with IgM suppression. However, it would be technically challenging to test this hypothesis in San Francisco given the relative absence of high peak pollutant levels.

As expected, smoking appeared to interact with PM_10_ and NO_2_ to augment suppression of serologic responses to Msg, a finding supported by prior studies. For instance, Crothers et al., using similar recombinant Msg and ELISA protocols, recently found that, among a cohort of active heavy smokers, smoking was associated with decreased serologic responses to *Pneumocystis* Msg compared with non-smoking controls [36]. Many of the pollutants in cigarette smoke are similar to those in ambient air pollution, but at much greater concentrations. Given these properties, we expected smoking to also be independently associated with suppressed IgM, which it was not in this study. It was the smoking—AAP interaction, and not smoking alone, that was associated with suppressed IgM responses. Why we failed to find an independent effect of smoking in this study is unclear.

Although cellular immune responses are predominantly involved in effectively countering *Pneumocystis jirovecii* infections, it is well known that humoral immunity plays an essential role [37-46]. For instance, B cell deficient mice succumb to *Pneumocystis* infection much more rapidly than their immunocompetent controls [41]. In humans, cellular immunosuppression, caused by immunosuppressive medications and conditions such as HIV infection, play a prominent role in the increased risk for PCP, but deficiencies of humoral responses, such as congenital X-linked agammaglobulinemia, may also impart an increased risk [47,48]. Given the known importance of humoral immunity of *Pneumocystis* infection in animals and humans, we hypothesized that ambient air pollution exposures, mediated by suppressed IgM responses to *Pneumocystis* Msg, would be associated with adverse clinical outcomes. Although we found a trend for decreased IgM responses associated with increased length of hospital stay, the precise reasons for this finding are unclear as length of stay is influenced by numerous factors. IgM responses were not associated with the other measured clinical outcomes, nor were ambient air pollutants associated with clinical outcomes. It is important to note that the number of clinical endpoints in our study were too few to adequately test this clinical hypothesis.

Our study has several limitations. Ambient air pollution exposures were estimated from daily levels measured by the single EPA compliance air quality monitoring station in San Francisco. Fortunately, the majority of our study participants resided within a 3 km radius of this station such that using the air quality data from this station to estimate subject exposures was reasonable. Our sample size was small, and we were unable to include a number of patients with confirmed PCP due to lack of serum specimens drawn within 2 days of admission. However, we are confident that the excluded cohort did not significantly bias our study findings or alter our conclusions given the similar clinical characteristics and comparable clinical outcomes among included and excluded cohorts. Finally, San Francisco experiences relatively low levels of all the criteria ambient air pollutants. Perhaps we would have found more pronounced immunotoxic effects and clinical outcomes in a more polluted city.

In conclusion, elevated 3-day average PM_10_ and NO_2_ ambient air concentrations immediately prior to hospital admission and 2 weeks prior to admission were associated with suppressed IgM antibody responses to recombinant *Pneumocystis jirovecii major* surface glycoprotein in HIV+ patients hospitalized with confirmed PCP, and active cigarette smoking appeared to augment the immunosuppressive effects of these pollutants. These provocative preliminary findings suggest a mechanism of immunotoxicity by which ambient air pollution increases host susceptibility to pulmonary infection. A larger prospective cohort study with more precise individual-level exposure assessments and evaluating both IgM and IgG responses is needed to confirm these findings and to better assess their clinical significance.
